# Needs, Modes, and Stances: Three Cardinal Questions for Psychotherapy Practice and Training

**DOI:** 10.32872/cpe.12753

**Published:** 2024-04-26

**Authors:** Eshkol Rafaeli, Alexandra K. Rafaeli

**Affiliations:** 1 Department of Psychology and Gonda Center for Neuroscience, Bar-Ilan University, Ramat-Gan, Israel; 2 Unit for Psychological Services, Ben-Gurion University, Beer-Sheva, Israel; Department of Psychology, University of Trier, Trier, Germany

**Keywords:** process-based therapy, universal psychological needs, modes/self-states, therapeutic stances, psychotherapy integration

## Abstract

**Background:**

Advances in motivational science (Dweck, 2017), personality dynamics (Lazarus & Rafaeli, 2023), and process-based psychotherapy (Hofmann & Hayes, 2019) converge into a pragmatic, integrative, and transtheoretical model of practice and training.

**Method:**

The model comprises three elements: a formulation centered on clients’ psychological needs which provides guidance regarding the goals and processes most profitable to pursue; a recognition that such pursuit frequently requires contending with a multiplicity of clients’ internal self-states (i.e., modes); and an enumeration of pragmatic therapeutic stances likely to help address clients’ need-related goals in light of their modes.

**Results:**

We distill these elements into three cardinal questions: What needs does this client have that are not currently met, and what are the most profitable ways of remedying that frustration? What mode or modes does this client manifest – both generally and at this very moment? and What stance should I adopt in response to the client’s current mode? We suggest that clinicians should be trained to continually pose these questions and seek to answer them collaboratively with their clients.

**Conclusion:**

This model – illustrated here using schema therapy terms – offers a process-based approach which serves as a theoretically integrative starting point but is general enough to provide an assimilative integration roadmap for therapists anchored in most primary orientations. Integrative or assimilative therapists trained to attend to needs, modes, and stances are likely to be (and be perceived as) particularly responsive, and thus, to enact “common factor” practices known to be conducive to therapeutic alliance and gains.

## Highlights

- We present three crucial tasks for facilitating effective clinical training and practice.
- First, identify core psychological needs to set therapy goals and effective interventions.
- Second, recognize the multiplicity of clients' different self-states (or modes).
- Third, flexibly adopt specific stances to respond well to clients' needs and modes.

People seek and enter psychotherapy because something is amiss. It is the role of psychotherapists to help formulate what that “something” is, translate it into identifiable goals for change, present possible pathways towards these goals, collaborate with their clients as they try out these pathways, respond to unforeseen obstacles and turns-of-events along the way, and ultimately help their clients get their frustrated or thwarted needs more adequately addressed.

In this article, we will argue for a model of practice (and thus, of training) that can help psychotherapists embody and enact this role well. This model emphasizes three key elements. The first element is a formulation of a client’s story (or their presenting complaints) in terms drawn from *a universal taxonomy of psychological needs*, which provide guidance regarding the goals and processes that would be most profitable to pursue in therapy. The second element is the recognition that such pursuit does not always follow a straight path, and often involves grappling with *a multiplicity of clients’ internal self-states* (or *modes*). The third element, which builds on these first two, is an enumeration of pragmatic *therapeutic stances* which help address the client’s need-related goals in light of their modes. In concluding, we will put forward the idea that therapists trained to attend to *needs*, *modes*, and *stances* and to make implicit – or better yet, explicit – use of these elements are likely to be (and to be perceived as) particularly responsive, and thus, to enact “common factor” practices known to be conducive to therapeutic alliance and gains.

One note: the model we present is drawn from schema therapy (Rafaeli et al., 2010; Young et al., 2003), a theoretically integrative approach rooted in cognitive behavioral, psychodynamic, and experiential thinking. Indeed, throughout the paper, we use schema therapy terms to illustrate the three elements discussed. However, as we hope to show, the key points presented here do not require one to subscribe to schema therapy per se, or even to favor *theoretical integration*. Instead, they may provide a roadmap for *assimilative integration* for therapists anchored in most primary orientations.

**Let’s begin with the first element.** Recent years have brought a growing understanding that empirically supported treatments have probably gained much of what there is to gain from categorical identification of syndromes and from the development of syndrome-specific protocols or interventions (Hofmann & Hayes, 2019; Insel, 2022). In their stead, the time seems ripe for process-based therapy – interventions that eschew diagnostic labels and focus instead on procedures most likely to change underlying biopsychosocial processes or mechanisms tied to desirable treatment outcomes or goals across diagnostic boundaries.

But how should these processes or mechanisms be identified? We would argue that a straightforward taxonomy of change mechanisms, one that is most likely to make intuitive appeal to clients as well, is ***a taxonomy of universal psychological needs***. After all, as our opening paragraph illustrated, the logical transition from saying that people enter therapy “because something is amiss” to viewing it as an issue of addressing “frustrated or thwarted needs” is quite seamless.

In a way, most schools of psychotherapy have an explicit or implicit model of *motives* or *needs* at their core. Some base this core, explicitly, on Darwinian evolutionary principles (Gilbert, 2019; Hayes et al., 2020) and note the centrality of the broad needs for *survival* and *reproduction*, from which they draw more specific goals (e.g., social safeness; Gilbert et al., 2008). Others adopt the principles of Bowlby’s attachment theory (e.g., Davila & Levy, 2006; Goodwin, 2003; Slade & Holmes, 2019), itself strongly influenced by Darwinian thinking, and focus on specific biobehavioral drives towards *pair-bonding*, *care-giving*, and (centrally) the formation and maintenance of *attachment* bonds. (Notably, a wide range of approaches use the term “attachment” in their title, or identify it explicitly as a key part of their model; e.g., Diamond et al., 2003; Hughes, 2004; Johnson, 2019; Milrod et al., 2016; it’s interesting to consider why the same credit hasn’t been given to Darwinian theory). And of course, many approaches to psychotherapy – especially those that gained prominence in the mid 20^th^ century – place particular (explicit) premia on needs that may be more uniquely human: e.g., the need for authenticity (Perls et al., 1951), meaning (Frankl, 1959), self-actualization (Rogers, 1963), and creativity (May, 1969).

For various reasons (probably similar that those recognized in adjacent fields, like personality psychology; e.g., Del Giudice, 2018; Zeigler-Hill et al., 2019), motivational accounts within psychotherapy fell out of favor in the height of the cognitive revolution of the 1970s-1990s. A possible consequence has been that evidence-based psychotherapy approaches which came of age in those decades – including ones with which we strongly identify (e.g., cognitive behavioral therapy; Barlow, 2021; Beck, 1970; interpersonal psychotherapy, Markowitz & Weissman, 2004) have stayed rather silent when it comes to discussing needs or other motivational constructs. This does not mean that motives or needs – e.g., for safety, connection, competence, or even simply for a world that can be adequately understood – aren’t implicitly present in these therapeutic models. It just means that they are not seen as key concepts within these approaches. **Thus, our first proposed element is that the practice of psychotherapy – and training in it – should adopt an explicit and transtheoretical language to describe psychological needs so as to help therapies achieve their most basic goal of addressing these needs.**

What should this language be? Rather than pitting one theoretical school (say, humanism) against another (say, attachment-based or evolutionary-based approaches), we would argue that psychotherapists should instead follow the lead of pioneers such as Grawe (1997) in turning to vibrant work being done in the broader field of psychology. Grawe turned to Miller et al.’s (1960) work on Plans to develop his Consistency Theory, which emphasized the role of need fulfillment in promoting well-being and facilitating positive therapeutic change. Today, we can build on more modern motivational work, in which recently developed frameworks (e.g., Del Giudice, 2018; Dweck, 2017; Schaller et al., 2017) still lead to remarkably similar clinical conclusions.

We’ll illustrate this with one particularly comprehensive framework – Carol Dweck’s (2017) recently-proposed model linking motivation, personality, and development. Dweck's model synthesizes extensive literature on psychological needs from both basic and clinical research to provide a broad and inclusive taxonomy of needs, including three basic ones and 4 compound ones. The three basic needs – for *acceptance/belonging, competence,* and *optimal predictability* (i.e., sufficient order and stability) – are thought to be universal, present at birth, and non-derivative. The compound needs for *control* (or *autonomy*), *trust*, and *status/self-esteem,* though also universal, are thought to each emerge a bit later in development from the conjunction of two basic needs (e.g., *trust* integrating *acceptance* and *optimal predictability*) and to require meta-cognitive capacities not present at birth (e.g., self-awareness). Finally, the ultimate compound need for *self-coherence,* encompassing meaning and identity, is thought to be fed by all other compound as well as basic needs and to serve as the “master sensor” of whether things are as they should be.

Dweck (2017) argues that needs give rise to goals, and that as people pursue these goals, they develop representations (which she refers to as BEATS: Beliefs, representations of Emotions and representations of Action Tendencies). Understanding these needs and the ensuing BEATS is key to understanding human development, motivation, and personality. Importantly, it is also key to understanding human distress and it’s amelioration. Specifically, thwarted or frustrated needs and their down-stream consequences (namely, ineffective or maladaptive goals or representations) are key determinants of poor psychological well-being and should therefore be the focus of psychotherapy.

Most clinicians find this basic idea of putting *needs* at the forefront entirely consonant with the underlying assumptions driving their clinical work. Yet, with few exceptions (e.g., Consistency Theory: Grawe, 1997; motivational interviewing: Miller & Rollnick, 2002; Ryan et al., 2011; schema therapy: Rafaeli et al., 2010; Young et al., 2003), these assumptions typically remain silent, even when the therapy is guided by an otherwise explicit case conceptualization (see Gilboa-Schechtman, 2024, this issue). We argue that by offering (or at least attempting to develop) a comprehensive model of psychological needs, Dweck’s (2017) framework provides us with an approach for organizing any therapeutic work we do. This would be relevant in relatively straight-forward situations, in which one of the basic needs (for optimal predictability, belonging/acceptance, or competence) is unmet. And it would be even more relevant when later-appearing compound needs (for control/autonomy, trust, self-esteem/status, or self-coherence [i.e., meaning and identity]) are frustrated, or when multiple needs compete or become intertwined.

Indirect evidence that good therapy helps clients meet their needs, and thus, improve their ability to live meaningful, satisfying lives full of love and work (cf. Freud, 1930) abounds. But despite the intuitive appeal of this model, and despite calls for the actual assessment of need satisfaction or frustration (e.g., Vansteenkiste & Sheldon, 2006), limited empirical work to date has explored need-satisfaction directly. Even schema therapy, which expressly speaks about the recognition and importance of needs, rarely uses measures to directly assess need satisfaction or frustration.

Once needs are identified, understood, and explored, the *ends* (or “targets”) of therapy become much clearer. But what about the *means* to reach these therapeutic ends? With respect to this pragmatic question, the psychotherapy field is full of many effective/efficacious therapeutic interventions, drawn from diverse orientations that can help clients satisfy specific needs. For example, a frustrated need for competence is often profitably addressed using behavioral interventions such as graded task assignment; a frustrated need for relatedness is often addressed with interpersonal therapy interventions such as communication analysis; and a frustrated need for self-worth or self-esteem, likely to be accompanied by harsh self-criticism, may be most amenable to techniques such as two-chair dialogues, drawn from Greenberg's (2004) emotion-focused therapy, as well as to self-affirmation tools taken from Gilbert's (2014) self-compassion therapy.

The training implications of focusing on needs are clear: trainees should become familiar with need models and should be provided with at least a basic toolset of therapeutic interventions that could serve as “first-line” choices once a client’s core needs are identified. If we had to pare this entire element down to one supervisory point, it is that clinicians (and trainees) should strive to answer this first cardinal question: “*What need or needs does this client have that are not currently met, and what are the most profitable ways of remedying that frustration?*”.

Based on this logic, we (the first author together with Aaron Fisher at UC Berkeley and Gal Lazarus at the Hebrew University) are currently implementing a randomized clinical trial comparing brief intervention protocols that are personalized (or not) with respect to the client’s most glaring frustrated need. To do so, we adopted specific empirically-supported techniques from a variety of models deemed to be good first-line need-focused interventions (see Table 1 for our choice interventions). Whether these will indeed prove efficacious with respect to need fulfillment is of course an empirical question; if they do not, others will.

**Table 1:** A Listing of Psychological Needs, Characteristic Distress Tied to Their Frustration, and Suggested First-Line Intervention Tools for Each

*The need*	*The characteristic distress (and most prominent schemas)*	*High-likelihood first-line interventions (and the approaches from which they are drawn)*
*Optimal Predictability*	Worried, anxious (*Vulnerability to harm*)	Acceptance and commitment (ACT) or mindfulness tools for emotion regulation
*Acceptance/Belonging*	Lonely, rejected, isolated (*Social isolation, Abandonment*)	Interpersonal Psychotherapy (IPT) tools to create change in the interpersonal sphere
*Competence*	Dependent, incompetent (*Dependence, Failure, Insufficient self-control*)	Behavior therapy (BT) techniques to improve performance
*Trust*	Mistrustful, hurt (*Mistrust/abuse, Emotion deprivation*)	Schema therapy (ST) tools, such as imagery work on trust violations
*Autonomy/Control*	Outwardly focused or unmotivated (*Subjugation, Enmeshment, Undeveloped self, Approval seeking*)	Assertiveness training tools, decisional balance chair-work, motivational interviewing tools
*Self-esteem/Status*	Ashamed, self-critical (*Defectiveness/shame, Unrelenting standards, Punitiveness*)	Self-compassion therapy (SCT) tools, emotion focused (EFT) tools for combatting the self-critic
*Self-coherence (meaning, identity)*	Lost, identity-less, nihilistic (*Self-sacrifice, Entitlement/Grandiosity*)	Values (commitment) work from ACT

**Which brings us to the second element.** As many clinicians, including novice ones, quickly learn, the picture of clearly defined aims (whether they be *fulfillment of frustrated needs* or *removal of diagnostic symptoms*) achieved through clearly defined means (including evidence-based ones, known to be effective on average), is an overly idealized version of many therapeutic processes. Even when clinicians ask the first cardinal question noted above and reach well-founded answers for it, they often run up against substantial obstacles which require attention to the here-and-now of therapy.

One example of these obstacles can be observed within the context of the therapeutic alliance. Extensive research on alliance (see Flückiger et al., 2018) attests to how important but non-trivial it is to establish therapeutic bonds, and to develop shared understanding of the goals to which therapy should aspire and the tasks that could lead there. But alliance fluctuates across therapists, clients, sessions, or even moments (Zilcha-Mano, 2021), and alliance ruptures, impasses, and interpersonal enactments are ubiquitous in therapy (Safran & Kraus, 2014). Why do these occur?

The state-like nature of alliance (and alliance ruptures, enactments, etc.) points to a likely culprit: namely, the fact that people themselves (including clients and therapists) are not fixed actors or agents, but rather a collection of multiple selves (Markus & Wurf, 1987), parts (Bromberg, 1996), “I-positions” (Hermans, 2001), modes (Rafaeli et al., 2016), or as Bill Stiles poetically noted – “a community of internal voices” (Stiles, 2011).

As this (very partial) list of terms illustrates, many clinical models (and increasingly, social, developmental, and personality research findings) seem to converge on a similar idea: that humans move around between different “modes” (our preferred term) – cohesive, experientially distinct, state-like manifestations of personality characterized by specific profiles of affects, behaviors, cognitions, and desires (Lazarus & Rafaeli, 2023). Explicit attention to these modes in theory and research – but also in clinical practice (Rafaeli et al., 2014; Ryle & Fawkes, 2007; Stiles, 2011) – can provide an organizing framework for understanding both "typical" personality and all (or at least most) forms of psychopathology. And as we’ll show in a minute, they also play an outsized role in the here-and-now of therapy. **Thus, our second proposed element is that practice and training of psychotherapy must prepare clinicians to see *multiplicity* within their clients (and themselves).**

The idea that modes are present to some degree in every person’s phenomenology is easily intelligible to most people, who know, viscerally, how different it feels to be hurt, angry, self-critical, detached, reflective, playful, and so on. But therapists and trainees who become attuned to such modes (or voices, or selves, or parts, etc.) can use this attunement to facilitate therapist-client communication, practice more effective empathy, and repair alliance ruptures more effectively. They can also have (and share with their clients) an experience-near understanding of the clients’ varying and often distressing psychological states.

Not every client requires mode-based work. Certainly, some clients enter therapy with sufficient reflectiveness and self-compassion (referred to, in schema therapy, as a “healthy adult mode”; Young et al., 2003) and/or with sufficient playfulness and creativity, so that even if they do manifest some vulnerable modes (marked by pain or distress), they are relatively unencumbered by introjected voices (marked by self-criticism or self-punishment) or maladaptive coping parts (marked by avoidance, surrender, or over-compensation). With such clients, answering the first cardinal question posed earlier could suffice; after the focal needs are correctly identified, and a well-suited targeted response implemented, we should see their pain or distress abate. For example, a client with a relatively simple dilemma regarding a specific life decision may be accurately seen as lacking in (say) *autonomy* or *internal motivation;* these could probably be augmented by using (say) *assertiveness training, decisional balance,* or *acceptance-and-commitment tasks*.

Often, however, broad personality traits and/or more specific pernicious modes (which may reflect such traits) are so prominent that therapy invariably must address them or at least take them into account. For example, trait *perfectionism* (e.g., Zinbarg et al., 2008), as well as state or trait *self-criticism* (e.g., Löw et al., 2020; Werner et al., 2019) have been tied to poorer treatment response; the same is true for *avoidance* (e.g., grosse Holtforth, 2008). To address these, therapists should be prepared to ask a second cardinal question: “*What mode or modes does this client manifest – both generally and at this very moment?*”.

Answering this question helps conceptualize the client’s presentation in mode terms and brings this awareness of modes into clinical work. Mode-aware clinical work (e.g., Rafaeli et al., 2014; Ryle & Fawkes, 2007; Stiles, 2006) aims to achieve better integration among modes through three broad processes: identifying and labeling individuals’ notable or recurrent modes; giving voice to adaptive and vulnerable modes over maladaptive or introjected ones; and creating adaptive boundaries between modes in ways that alter the relative dominance or power of specific modes. These processes frequently involve psychoeducation about the universality of modes coupled with cognitive, behavioral, and experiential methods. Quite often, though, the best way to advance these processes is by implementing **the third element of our model – the idea that mode-aware clinical work calls for the flexible adoption of different *therapeutic stances* depending on the client’s active mode.**

Explicit or implicit *therapeutic stances* are present in many clinical approaches, and function as general rules for how therapists using *that* particular approach should engage with their clients. Examples include using an open/accepting stance in acceptance and commitment therapy (e.g., O’Neill et al., 2019), engaging in collaborative empiricism in CBT (Tee & Kazantzis, 2011), maintaining neutrality in transference-focused therapy (e.g., Clarkin et al., 2021), experiencing and expressing empathy in self-psychology (e.g., Kohut, 1981/2010), etc.

Though each of these stances may have its merits, they are often contradictory (e.g., acceptance vs. change, neutrality vs. empathy); moreover, there’s little reason to think that any one stance necessarily fits all clinical circumstances. Rather than adhering to a single therapeutic stance, therapists attentive to modes have the opportunity to address their clients differentially – i.e., to ask themselves the third cardinal question: *“what stance should I adopt in response to the client’s current mode?”*

To illustrate this idea, we consider the recommendations made within schema therapy (e.g., Rafaeli et al., 2014) regarding the stances that would be most effective vis-à-vis different categories of client modes (see Table 2). We use these schema therapy categories because we see them as striking a good balance between optimal distinctiveness (i.e., minimizing definitional overlap among modes) and parsimony (i.e., limiting the number of modes as much as possible); for more discussion on adjudicating the number and identity of modes or mode categories, see Lazarus and Rafaeli (2023).

**Table 2:** Mode Categories, Relevant Example Modes, and Suggested Therapeutic Stances

*Mode Category*	*Relevant Mode(s) in Schema Therapy*	*Suggested Therapeutic Stance*
*Reflective and Self-compassionate Mode*	Healthy Adult	*Joining* and mirroring this mode’s behavior when it is present. *Modeling* such behavior when it needs strengthening.
*Child Modes (i.e., basic emotional need states)*	Distressed Mode, Vulnerable Child, Angry Child	Using *limited reparenting* to directly meet client emotional needs (e.g., appropriate nurturance, protection, limit-setting, encouragement, playful joining).
*Maladaptive Coping Modes*	Detached/Avoidant Protector, Hopeless Surrenderer, Perfectionistic Over-controller, etc.	Using the dialectic stance of *empathic confrontation* (empathy plus confrontation of maladaptive behaviors).
*Dysfunctional Introjected Voices*	Self-critic, Punitive Parent Mode, etc.	Using straightforward *confrontation* to make the voice ego-dystonic; Siding with the Healthy Adult against it; Providing *psychoeducation* in less severe instances.

Let’s begin with the simplest stance, relevant to moments in which clients present with a strong, reflective (“Healthy Adult”) mode. In such moments, therapists are free to employ various evidence-based tools (and possibly adopt therapeutic stances such as CBT’s collaborative empiricism or ACT’s openness and acceptance). In broader terms, the therapeutic stance can be thought of as *joining* and as *modeling* of adaptive problem solving.

In other moments, clients’ activated modes clearly reflect basic emotional need states: *distress* of various sorts (which schema therapy refers to as the “Vulnerable Child”), *anger* over unmet needs (“Angry Child”), *impulsivity* (“Impulsive Child”), but also *contentment/play* (“Content/Happy Child”). Schema therapy’s reference to these as “child modes” is meant to evoke the idea that when clients (of whatever age) are in such modes, the most viable and appropriate response is often to try and meet the emotional needs directly – within the ethical boundaries of therapy – in ways that simulate a good-enough parent or attachment figure. Thus, activated modes marked by intense primary emotions occasion a therapeutic stance referred to as *limited reparenting*, which aims to address the client’s hyper-arousal, help soothe them back into a window of tolerance (Ogden & Minton, 2000), and (ultimately) serve as a model for healthy self-care. Intriguing evidence in support of this idea comes from recent work (Fisher et al., 2023) showing that therapists’ oxytocin responses (a marker of caregiving system activation) following genuine displays of client distress mediate the association between client negative emotion activation and symptomatic change.

Quite often, clients manifest maladaptive coping modes, in which they attempt – knowingly or not – to avoid the distress of unmet needs using coping behaviors (e.g., avoidance, over-compliance, over-compensation) and/or cognitions (e.g., detachment, surrender, self-aggrandizement). These may bring short-term relief, but come with a hefty long-term cost: coping modes impede both raw emotional need states (i.e., the Vulnerable Child mode which evokes limited reparenting) and healthy (i.e., reflective, compassionate) states that would permit real engagement. To address coping modes, ST calls for a third therapeutic stance, a dialectic balance of empathy and confrontation referred to as *empathic confrontation*. In this stance, therapists strive for *empathy* (or at least curiosity) for the need itself, for the distress that accompanies its frustration, and for the short-term relief brought about by the coping behaviors/cognitions; at the same time, they directly *confront* the specific behaviors or cognitions which are deemed maladaptive, and help clients develop healthier alternative behaviors/cognitions.

Finally, therapists may come face-to-face with clients’ introjected voices (e.g., voices of parents, other significant others, peers, or the society at large) that are a root cause of distress. These include punitive, self-critical, neglectful, and self-denigrating voices, and they call for yet another stance: one in which the therapist sets limits or directly confronts the introjects to help clarify their ego-dystonic nature and thus weaken them.

The terminology and clinical guidelines above are drawn from schema therapy (e.g., Rafaeli et al., 2016), an approach documented by a growing body of research to offer effective treatment for a range of relatively complex and chronic conditions (e.g., Peeters et al., 2022; Zhang et al., 2023). Other mode-aware approaches may delineate the modes somewhat differently (e.g., Stiles, 2006) or offer somewhat different guidelines for choosing differential stances to address them (e.g., Fosha, 2000; Gilbert, 2019; Greenberg, 2004). Arbitrating which of these stances would work best remains an open empirical issue.

**Conclusion.** We presented a model of practice and training containing three elements, translated into the three cardinal questions: “What *unmet needs* are most prominent for this client and how could they be addressed?”, “What *mode* is the client in right now?”, and “What *stance* would work best to address this client’s needs while in this mode?”. These elements distill integrative ideas from schema therapy (Rafaeli et al., 2010; Young et al., 2003), but we believe they are general enough to serve as a starting point for a unifying language for most, if not all, therapists – and for clients. Specifically, though cognitive-behavioral, emotion-focused, and psychodynamic therapists may quibble about the etiology of distress, the pros and cons of alleviating distress through direct intervention (i.e., meeting vs. frustrating needs), or the merits of adopting flexible therapeutic stances, they are less likely to find cause for disagreement regarding the existence – and importance – of the first two elements discussed: *needs* and *modes*.

Establishing a formulation based on needs and (when needed) on modes as an explicit starting point, and using intuitive and experience-near terms to share this formulation with one’s client, empowers the client to have greater agency within their therapy. It also sets the stage for therapeutic work that harnesses contemporary understanding about both specific and common/nonspecific factors that exert beneficial therapeutic effects (see Hofmann & Barlow, 2014). Specifically, therapists attentive to the three elements described here are likely to be implementing transtheoretical “common factors” known to be conducive to therapeutic gains.

Consider the widely-studied common factor of therapeutic alliance, responsible for a substantial portion of therapy’s benefits (e.g., Flückiger et al., 2018). Two strong (though understudied) predictors of alliance are responsiveness (Reis & Gable, 2015) and high-quality listening (Itzchakov et al., 2022). Both involve getting a clear picture of the client’s real as well as perceived needs (Refoua & Rafaeli, 2023), and being attuned to their present "mode". Therapeutic alliance, and repair of ruptures in this alliance, require such responsiveness. Specifically, in addressing clients’ core needs, a responsive therapist needs to identify whether, at the moment, they are mostly overwhelmed with pain/sadness/fear and can (at most) absorb some comfort (i.e., limited reparenting); too defended (detached, avoidant, compliant, argumentative, over-controlling, etc. – i.e., in a coping mode) to do any productive work and need to be coaxed away from these coping modes into more productive modes; truly toxic towards themselves (i.e., in an introjected negative mode) and need some limit-setting; or else are present, reflective, integrative, and self-compassionate enough (i.e., in a healthy adult mode) and thus able to work, shoulder-to-shoulder, towards their goals.

Interestingly, transtheoretical work on rupture resolution (e.g., Safran et al., 2011) touches on these three elements as well. In particular, it recognizes that different *needs* (e.g., for communion vs. agency) may underlie disagreements regarding tasks or goals, and/or deterioration in the therapeutic bond. Additionally, it adopts a similar “what to do when” approach, and thus calls for specific *stances* in response to different *states*. Interestingly, whereas rupture repair work focuses on alliance states and views them predominantly intersubjectively, our model allows for, but does not assume, such intersubjectivity.

Of course, as the reviewers of an earlier draft correctly noted, even if we as clinicians and supervisors converge on this universal starting point of needs, modes, and stances, many questions remain to be explored: What instruments or procedures should be used to assess these? Should interventions to address specific needs be selected primarily based on empirical evidence or guided by theoretical principles? Should these choices be made uniformly across all therapeutic schools, or should we expect variation in the selection process within these schools? And would prospective trainees be expected to develop expertise in all distinct first-line interventions and/or in all the approaches from which they are drawn?

**Coda.** The approach outlined here is decidedly integrative in several respects (Castonguay et al., 2015). It can serve as a *theoretically integrative* starting point; it adopts *technical eclecticism* in addressing specific needs (see Table 1); it speaks (as we’ve shown) to the issue of *common/nonspecific factors*; and it is general enough to provide those clinicians who choose to remain anchored in a primary orientation (be it CBT, experiential, dynamic, or systemic therapy) a roadmap for *assimilative integration* of concepts that may not be endemic to their approach, but are also not likely to be too foreign. After all, the elements presented here tend to be consistent with most people's (including clinicians’) lay understanding of what distress is about (i.e., unmet needs) and of what phenomenology is about (i.e., "parts" or "selves" or "modes"). The model includes a healthy dose of therapist humility and is deliberately jargon-free. Consequently, it can help engage clients in collaboratively posing and answering the cardinal questions presented, developing a shared language for talking about their needs, goals, and modes, and ultimately reaching desired outcomes.
